# The role of vitamin D receptor and IL‐6 in COVID‐19

**DOI:** 10.1002/mgg3.2172

**Published:** 2023-04-06

**Authors:** Ali Azmi, Maziyar Rismani, Hossein Pourmontaseri, Ebrahim Mirzaii, Sedigheh Niknia, Behnoosh Miladpour

**Affiliations:** ^1^ Student Research Committee, Fasa University of Medical Sciences Fasa Iran; ^2^ Department of Biochemistry Fasa University of Medical Sciences Fasa Iran; ^3^ Bitab Knowledge Enterprise, Fasa University of Medical Sciences Fasa Iran

**Keywords:** cytokine, gene expression, interleukin‐6, SARS‐CoV‐2, vitamin D receptor

## Abstract

**Background:**

Vitamin D (Vit.D) has an important role in protecting COVID‐19 patients. This study investigated the changes in vitamin D receptor (VDR) expression and interleukin 6 levels in patients with COVID‐19.

**Materials and methods:**

120 hospitalized patients and 120 healthy people participated in this study, both group adjusted by sex and age. Vit.D was measured with HPLC, the expression of VDR gene was done with Real‐time PCR, and IL‐6 was measured with ELISA assay.

**Results:**

Our findings showed no significant difference in the case of Vit.D (25‐OH‐D3) between the two studied groups, interestingly the expression of VDR was statistically lower in the patients with COVID‐19, *p*‐value = 0.003. VDR expression was lower in the patient with diabetes, hypertension and cardiovascular disease, significantly, *p*‐value = 0.002. The level of IL‐6 was statistically higher in the COVID‐19 group, *p*‐value = 0.003.

**Conclusion:**

Alongside the important role of 25‐OH‐D3 in COVID‐19 patients, the quality and quantity of the VDR expression and its role in the level of IL‐6 are the promising risk factors in the future. Further studies are needed to determine the factors increasing the expression level of VDR, especially in the patients with diabetes, hypertension and cardiovascular disease.

## Graphical abstract

Vitamin D receptor expression and interleukin‐6 serum levels in patients with covid‐19

There was not any significant difference between COVID‐19 patients and the control group (people whose COVID‐19 test was negative) in serum levels of 1,25 OHD3.
The expression of Vitamin D receptors was significantly lower in the COVID‐19 patients compared to the control group.
The expression of Vitamin D receptor was significantly lower in COVID‐19 patients, with high BMI, and hypertension.
IL‐6 was significantly higher in the patients with COVID‐19.

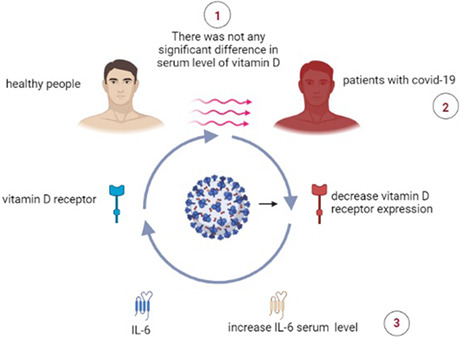

## 1 INTRODUCTION

In December 2019, a new coronavirus was discovered in Wuhan named coronavirus 2019 (COVID‐19) by World Health Organization (WHO). COVID‐19 spread worldwide in just four months and made the main pandemic of the century (Chang et al., 2020; Guan et al., 2020). The WHO recorded around 255 million confirmed COVID‐19 cases and more than 5.1 deaths associated with COVID‐19 (World Health Organization, 2021).

The severity of COVID‐19 is associated with several supplementation disorders, such as vitamin D deficiency (VDD) (Pereira et al., 2020). Ultraviolet rays of the sun stimulate the conversion of vitamin D3 to 7‐dihydro calcium thiol under the influence of enzymatic reactions in the skin. Then, vitamin D (Vit.D) reduces the severity and mortality of viral diseases using different mechanisms, including physical barriers, cellular natural, and adaptive immunity (Rondanelli et al., 2018). Viruses usually cause infections through disruption of cellular connections, but Vit.D prevents viral infections by maintaining these connections (Chen et al., 2020). Insufficient level of Calcifediol [25‐(OH)‐D3] and dysfunction of the immune system and adequate cellular response to Vit.D caused by microorganisms result in Vitamin D deficiency (VDD) and increase the severity of COVID‐19 (Ali, 2020). Animal studies have shown that injecting 4000 IU/d of Vit.D significantly inhibits the growth of the virus in tissues. Therefore, Vit.D supplementation and the level of 25‐OH‐D3 would play one of the most important determinant roles in the outcomes of COVID‐19 (Martínez‐Moreno et al., 2020).

Vit.D receptor (VDR) is another important factor in immunologic mechanisms that regulates T cells differentiation and activity stimulating Vit.D effects (Charoenngam & Holick, 2020). The Vit.D/VDR signaling pathway provides several promising influences in bacterial‐induced acute respiratory distress syndrome (ARDS) through different mechanisms, including regulation of the renin‐angiotensin pathways, restriction of cytokine storms specially IL‐6, a key interleukin in viral replication, protection of physical barrier in pulmonary epithelial, and stimulation of neutrophils (Xu et al., 2017; Zheng et al., 2020). Subramanian et al. showed that Vit.D decrease the production of proinflammatory cytokines like IL‐6, IL‐8, and IL‐12, and increase IL‐4 as an anti‐inflammatory cytokine in neutrophils that infected with pneumococci. Vitamin D lowers IL‐6 production by immune cell, and reduce proinflammatory effects (Subramanian et al., 2017).

The 1,25‐dihydroxy vitamin D3 [1,25‐(OH)2‐D3] activates the VDR, and the receptor binds to more than 23,000 genomic sites in different cells and results in the therapeutic effects of Vit.D (Rhodes et al., 2021). Moreover, VDR upregulates the conversion of 25‐OH‐D3 to 1,25‐(OH)2‐D3. Therefore, animal models with deficiency of VDR‐expressing gene cannot produce invariant NKT (iNKT) cells and CD8αα/TCRαβ T cells. As a result, due to the vital role of these two cell lines in the antiviral immune mechanism, these models suffer from many immune disorders (Kongsbak et al., 2013). Although some observations indicated that oral administration of 25‐(OH)‐D3 would improve the COVID‐19 severity but the therapeutic outcomes tightly depend on VDR expression (Quesada‐Gomez et al., 2020). One theory is that the polymorphism of VDR genes could be closely linked to comorbidities in COVID‐19 patients and ultimately determine the severity and mortality (Abdollahzadeh et al., 2021). Accordingly, the treatments that increase VDR expression have been highly welcomed to ameliorate the COVID‐19 outcomes (Evans & Lippman, 2020; Kongsbak et al., 2013). According to the critical role of Vit.D and its receptor in the immune response to viral infections, the present study compared the expression of VDR among COVID‐19 patients and healthy individuals. Also, the present study evaluated the difference in IL‐6 levels between COVID‐19 patients and the healthy groups due to its role in the inflammatory process during viral infections.

## 2 METHODS AND MATERIALS

### 2.1 Subjects and study design

#### 2.1.1 Editorial policies and ethical considerations

Our study was approved by the ethics committeof the Fasa University of Medical Sciences as IR.FUMS. REC.1399.075. The aim and procedure of the study were explained clearly to all participants and they were asked to complete the written informed consent. This cross‐sectional study was done including 120 COVID‐19 volunteer from Vali Asr hospital, Fasa University of Medical Sciences, and 120 healthy people. Both of the COVID‐19 group and healthy control group were matched with age and sex.

### 2.2 Inclusion criteria

New cases, adult individuals (>18 years old) of both sexes were included in the study. Among the individuals referred to Valiasr Hospital of Fasa, the participants with laboratory‐confirmed SARS‐CoV‐2 and mild or moderate clinical manifestations were defined as patients and recruited in the COVID‐19 group. The participants with negative SARS‐CoV‐2 test and no clinical symptoms related to respiratory infection were considered as the healthy control group.

### 2.3 Exclusion criteria

The participants with a history of serious comorbidities (including cancers, respiratory disorders, gastrointestinal disorders, and acute or chronic kidney disease), pregnancy, Vit.D supplementation or history of supplementation with immunosuppressive drugs were excluded. All the characteristics were collected through a self‐report questionnaire.

### 2.4 Measurements

Serum samples isolated from whole blood from COVID‐19 group and healthy control group were used for analyzing 25‐OH‐D3 and IL‐6. The expression of vitamin D receptor was investigated using RNA extracted from whole blood of COVID‐19 group and healthy control groups. The level of 25‐OH‐D3 was measured by HPLC. The serum samples were centrifuged, harvested, and stored in 2 aliquots at −20°C until the measurement process (1.5 mL micro‐centrifuge tube each). Until molecular assay, the whole blood specimens (2 mL) were stored in EDTA evacuated tubes at −20°C. The assay was done on Agilent 1260 HPLC instrument (5301 Stevens Creek Blvd Santa Clara, California 95,051, USA) with photodiode array detector set at reversed‐phase Agilent column (Microsorb‐MV 100‐5 C18, 250 × 4.6 mm, 5 μm) and 265 nm, respectively (Hanna et al., 2021). RNA purification from the whole blood samples was done using TRI Reagent Sigma Aldrich, Switzerland. cDNA synthesis was performed by cDNA synthesis kit (addbio, Korea). Then, real‐time PCR with sequence‐specific primers was used to define the VDR gene. In the end, the assay was done on stepOne™ (Applied Biosystems California 94,404 Foster City, USA) real‐time PCR. The serum level of IL‐6 was measured using Quantikine®ELISA, Human IL‐6 Immunoassay, CATALOG # D6050, according to the protocol described in the kit.

### 2.5 Statistical analysis

The Chi‐squared test was applied to compare demographic characteristics and 25‐OH‐D3. Also, linear regression was used to investigate the difference of VDR gene expression among two COVID‐19 group and healthy control group. All reported *p*‐values were compared based on a significance level of 0.05. SPSS® 17 software (SPSS Inc., Chicago, IL, USA) was used for all statistical analyzes.

## 3 RESULTS

Initially, 120 patients (mean age of 38.3 ± 15.4 years; 48 females) and 120 healthy participants (mean age of 37.7 ± 16.2 years; 54 female) were identified in the present study. Also, no significant differences in demographic features and plasma level of 25‐OH‐D3 were observed between the COVID‐19 group and healthy control group (Table 1).

**TABLE 1:** Demographic characteristics and 25‐OH‐D3 (ng/mL) of COVID‐19 group and healthy control group.

	Healthy group	COVID‐19 group	*p*‐value
Sex			
(Female)	54 (45%)	48 (40.0%)	0.726
(Male)	66(55.0%)	72 (60.0%)	
Age	37.7 ± 16.2	38.3 ± 15.4	0.881
25‐OH‐D3	23.4	24.1	0.670

The unadjusted model based on linear regression shows that the expression of VDR was significantly lower in covid‐19 group compared to healthy control group (*p*‐value = 0.003). Also, further models adjusted with sex, age, DM, and CVD showed higher significance (*p*‐value = 0.002). Table 2 shows the applied models of the present study.

**TABLE 2:** Comparing the expression of vitamin D receptor between COVID‐19 group and healthy control group.

Model	*R* ^2^	Unstandardized coefficients		*p*‐value*
		*B*	Std. error	
Model 0	0.173	−3.465	1.093	0.003*
Model 1	0.180	−3.498	1.114	0.003*
Model 2	0.203	−3.714	1.155	0.002*

* It means the *p*‐value is lower than 0.05 and statistically significant. Model 0, unadjusted; means VDR gene expression is significantly lower in the COVID‐19 group than healthy control group. Model 1, adjusted by sex and age; means after removing the confounders of age and sex, a significant difference was observed between the COVID‐19 group and healthy control group, Model 2, adjusted by Model 1 + diabetes mellitus, hypertension, and cardiovascular, means significance increased (0.002), when we adjusted the level of VDR gene expression with diabetes, hypertension and CVD.

**FIGURE 1:**
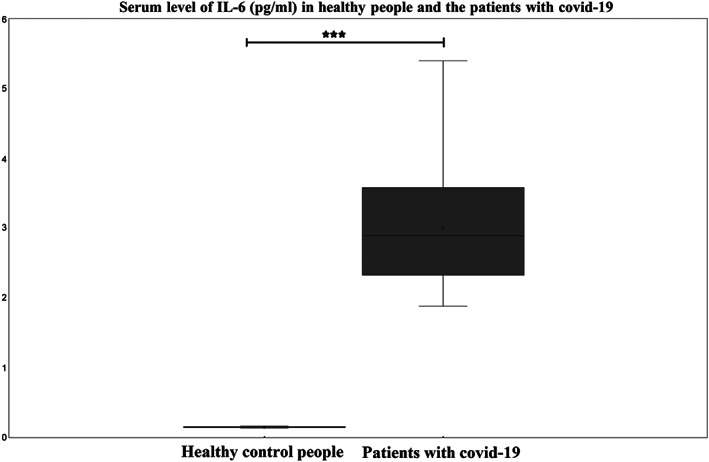
Comparison of serum level of IL‐6, between two COVID‐19 group and healthy control group. Serum level of IL‐6 showed significantly higher in COVID‐19 group than in healthy control group, the chi‐squared test, (*p*‐value = 0.001).

Measurement of serum levels of IL‐6 showed that significantly higher in the COVID‐19 group than in healthy control group, the chi‐squared test, (*p*‐value = 0.001), Figure 1.

## 4 DISCUSSION

The present study showed that vitamin D receptor (VDR) expression as well as IL‐6 levels were changed in patients with COVID‐19. Generally, our finding showed no statistically difference in serum level of 25‐OH‐D3 between COVID‐19 group and healthy control group. Interestingly, the expression level of VDR gene was dramatically lower in COVID‐19 group than healthy control people. We found that significance increased, when we adjusted the level of VDR gene expression with diabetes, hypertension and CVD. Also, we found that, serum level of the IL‐6 was statistically higher in the group of COVID‐19 patients than healthy control group. Vit.D would develop the immune system against infections through increasing the phagocytosis and mobility of macrophages (Evans & Lippman, 2020; Wu et al., 2019). Cytokine storm is a severe pulmonary pathology in which the widespread penetration of neutrophils and macrophages is facilitated, leading to large alveolar damage with the formation of hyaline membranes and diffuse thickening of the alveolar wall. Severe damage to the immune system and high serum levels of proinflammatory cytokines have been shown in patients (Huang et al., 2020; Qin et al., 2020; Xu et al., 2020). In particular, overproduction of IL‐6 is a valuable marker of poor outcomes in SARS‐CoV‐2 patients with acute respiratory distress syndrome (ARDS). Thus, cytokine induces systemic inflammation and endotheliopathy by inducing extensive secretion of inflammatory mediators from immune cells, activating complement components, increasing oxidative stress, and decreasing the expression of endothelial nitric oxide synthase, leading to damage to the heart, kidneys and multiple organs (Mokhtari et al., 2020). The COVID‐19 virus acts through the ACE2 protein. IL‐6 increases the levels of soluble adhesive molecules such as NLRP3 and ACE2r, which are important mediators in the effects of COVID‐19 on endothelial cells (Freeman & Swartz, 2020). Vit.D decreases the impact of IL‐6 through modulation of NF‐κB and STAT3 activation (Luo & Zheng, 2016). Since Vit.D bind to its receptors on the cell surface, enough expression of them in various organs of the body is very important in dealing with the COVID‐19 virus.

Many studies have shown the anti‐inflammatory effects of VDR, as a steroid hormone receptor family. The VDR has a critical role in alleviating inflammation by modulating the immune system. Infectious diseases are identified by triggering inflammatory genes such as nuclear factor kappa B (NF‐kB) that in turn activates IL‐6 and TNF‐α. In the immune system, VDR directly downregulates monocyte chemoattractant protein‐1 (MCP1) and interleukins like IL‐6, IL‐8, and IL‐1β, through interacting with VD‐responsive elements (VDRE) in regulatory genes. Meanwhile, VDR inhibits the proinflammatory nuclear factor κB (NF‐κB) pathway (Chen et al., 2013; Jonas et al., 2019; Nikniaz et al., 2021; Pojednic et al., 2015). In an In‐vivo study by Ami Febriza et al. on the role of IL‐6, TNF‐α, and VDR in the growth of Salmonella Typhi, they have shown that VDR level is inversely associated with the number of colonies of bacteria, on the other hand, the levels of IL‐6, TNF‐α, have direct communion with the numbers of colonies of bacteria (Febriza et al., 2020). In another study by P. McNally, they found that VDR and its agonists suppress proinflammatory cytokines production in cystic fibrosis (McNally et al., 2011). Bingwen Liu et al. have found in their study on COVID‐19 and inflammatory cytokines, that blockade IL‐6 may help to manage cytokine storm in patients. IL‐6 activates the signaling pathway of JAK (Janus kinase) by interacting with receptor IL‐6R, which directs to many biological effects ultimate to organ damage, such as converting naïve T cells into active T cells, the expression of vascular endothelial growth factor (VEGF) in epithelial cells, and increasing the permeability of the vessels (Liu et al., 2020).

Therefore, along with IL‐6 blockade drugs, pursuing the effective factors in the expression of VDR can be very helpful. Several studies have shown the role of SNPs in the expression of the VDR gene (Al‐Anouti et al., 2021; Li et al., 2021). Also, there is association between gene polymorphism of VDR and obesity (Rahmadhani et al., 2017). In our study, the number of obese people was limited; therefore, we could not investigate the relationship between BMI and the expression of VDR. In conclusion we found that the lower expression of the VDR and higher serum level of IL‐6 in the patients with COVID‐19 than healthy people.

## AUTHOR CONTRIBUTIONS

All authors contributed to the study conception and design. Study design, material preparation, data collection and analysis were performed by Behnoosh Miladpour. The first draft of the manuscript was written by Mziyar Rismani and Ali Azmi, experimental were performed by Sedigheh Niknia and Ebrahim Mirzaii and Hossein pourmontaseri. All authors commented on previous versions of the manuscript. All authors read and approved the final manuscript.

## FUNDING INFORMATION

This study was funded (NO: 97555) by Fasa University of Medical Sciences. Dr. Behnoosh Miladpour is the guarantor of this work and, as such, had full access to all the data in the study and takes responsibility for the integrity of the data and the accuracy of the data analysis.

## CONFLICT OF INTEREST STATEMENT

The authors declare no conflict of interest.

## Data Availability

All data will be available from corresponding author, with reasonable request.
